# Plasma Rich in Growth Factors (PRGF) in Transepithelial Photorefractive Keratectomy (TPRK)

**DOI:** 10.3390/jcm10091939

**Published:** 2021-04-30

**Authors:** José-María Sánchez-González, Federico Alonso-Aliste, Davide Borroni, Jonatan Amián-Cordero, Concepción De-Hita-Cantalejo, Raúl Capote-Puente, María-José Bautista-Llamas, María Carmen Sánchez-González, Marina Rodríguez-Calvo-de-Mora, Carlos Rocha-de-Lossada

**Affiliations:** 1 Department of Physics of Condensed Matter, Optics Area, University of Seville, 41012 Seville, Spain; mhita@us.es (C.D.-H.-C.); rcapote1@us.es (R.C.-P.); mbautista5@us.es (M.-J.B.-L.); msanchez77@us.es (M.C.S.-G.); 2 Department of Ophthalmology, Tecnolaser Clinic Vision, 41018 Seville, Spain; dr.alonsoaliste@tecnolasersevilla.es (F.A.-A.); dr.amian@tecnolasersevilla.es (J.A.-C.); 3 The Veneto Eye Bank Foundation, 30174 Venice, Italy; info.borroni@gmail.com; 4 Department of Doctoral Studies, Riga Stradins University, LV-1007 Riga, Latvia; 5 Department of Ophthalmology, Regional University Hospital of Malaga, 29010 Malaga, Spain; marocalmo@gmail.com; 6 Department of Ophthalmology (Qvision), Vithas Virgen del Mar Hospital, 04120 Almería, Spain; carlosrochadelossada5@gmail.com; 7 Department of Ophthalmology, University Hospital Virgen de las Nieves, 18014 Granada, Spain; 8 Department of Ophthalmology, Ceuta Medical Center, 51001 Ceuta, Spain

**Keywords:** plasma rich in growth factor, photorefractive keratectomy, transepithelial photorefractive keratectomy, visual outcomes, refractive outcomes

## Abstract

To evaluate the usage of plasma rich in growth factor (PRGF) in transepithelial photorefractive keratectomy (TPRK) in low and moderate myopia, patients who underwent myopic and astigmatism TPRK with PRGF were involved in this retrospective, observational study. Subjects underwent a surgical procedure between February 2019 and June 2019. A three-month follow-up was recorded. Pain score was assessed with a visual analogue scale (0–10) and re-epithelialization time recorded. A total of 48 eyes from 24 patients were recruited. Mean uncorrected distance visual acuity (UDVA) was 20/20.31 (0.00 ± 0.02 LogMAR). A total of 98% of eyes did not change corrected distance visual acuity (CDVA) lines. Two percent of eyes lost one line of CDVA. Preoperative spherical equivalent was −2.67 ± 1.37 D and after three months changed to −0.21 ± 0.34 D, and 2% of eyes changed 0.50 D or more between one and three months. Pain score was 3.29 ± 0.61 (3 to 6) score points at day one and 0.08 ± 0.27 score points at day seven. Finally, re-epithelialization time was 2.50 ± 1.20 days. PRGF addition to conventional refractive treatment such as TPRK seems to alleviate immediate postoperative pain and positively contribute to corneal re-epithelization time.

## 1. Introduction

Transepithelial photorefractive keratectomy (TPRK) was introduced by Gimbel et al. [1] in 1995. He used the excimer laser to eliminate the epithelium instead of the manual debridement. TPRK brought a series of innovations regarding alcohol-assisted photorefractive keratectomy (AA-PRK). No instrument contacts the eye during TPRK surgery and the surgery time is reduced. Also, the amount of the epithelial defect for ablation is more accurate than AA-PRK. Finally, the possible corneal toxicity due to the use of alcohol was eliminated [2]. The new generation of TPRK allows a customized and predefined amount of epithelial thickness to be removed. TPRK could be performed in a two-step transepithelial technique or in an advanced single-step TPRK combined with Smart Pulse technology. A recent meta-analysis demonstrated that TPRK is highly effective, safe, and predictable in correcting myopia and/or astigmatism [3]. However, not everyone in the ophthalmologist community make extensive use of the technique. The main reason is that there is no unified methodology. The classic TPRK works in two steps. In the first part, the epithelium is removed using a customizable uniform phototherapy keratectomy (PTK) to the central epithelial thickness [4] and subsequently PRK surgery occurs [5]. In addition, update TPRK procedure removes a customize epithelial layer using a population-based epithelial median profile. This epithelium was demonstrated not to have a uniform profile [4]. Furthermore, the two step procedure was not used worldwide due the long surgery time [6].

Autologous serum (AS), platelet-rich-plasma (PRP), and plasma rich in growth factors (PRGF) have been studied by Freire et al. [7] on human corneal epithelial cell with a follow-up of 24, 48, and 72 h. They concluded that substantially greater concentrations of epithelial growth factor were achieved in PRGF compared to AS and PRP. PRGF has been successfully used in recent ocular surface disorder investigations [8,9,10,11]. It has been used in chronic disease as a long-time period treatment, but also in short-time wound healing corneal disorders [8]. Recently, PRGF has been studied in the dry eye disease (DED) assessment and after laser-assisted in-situ keratomileusis (LASIK) [12], as an accelerator of the healing process in PRK [13] in mice [14] and as a regenerator of the corneal nerves after LASIK [15]. Other studies have analyzed autologous serum effect on re-epithelialization time in laser-assisted subepithelial keratectomy (LASEK) [16] and PRK [17].

The aim of our study was to assess the use of PRGF in TPRK recording the pain score and re-epithelialization time.

## 2. Materials and Methods

### 2.1. Design

This retrospective, observational study included forty-eight eyes from twenty-four patients who underwent myopic and astigmatism correction by TPRK with PRGF. Patients undertook surgical procedure among February 2019 and June 2019. All operations were completed at the Ophthalmology Center (Tecnolaser Clinic Vision^®^), Seville, Spain. Follow-up of all patients were three-months.

### 2.2. Ethical Aspects

All subjects involved in this research were effectively advised orally and on paper of the advantages, attributes, and dangers of the procedure. All patients authorized an informed consent previous to the surgical treatment and following the meeting done with the surgeon. This research was performed in agreement with the Helsinki Declaration tenets and data acquisition and analysis was performed in compliance with protocols approved by the Ethical Committee of University of Seville (ethical approval number 1141-N-16).

### 2.3. Subjects

Twenty-four patients (14 women and 10 men) freely attended private clinic to complete the exams and subsequently, when the surgeon verified their aptness for surgical procedure, they underwent myopic and/or astigmatism correction by TPRK with PRGF surgical treatment freely. Between the inclusion criteria: (1) a unchanging refraction for as a minimum of one year, means a variation ≤ to 0.50 diopters (D) in the refraction (spherical and cylindrical); (2) age between 21 and 40 years; (3) spherical equivalent myopia from −0.25 D and −7.00 D; (4) existence of astigmatism among 0.00 D and−2.50 D; (5) previous corrected visual acuity (CDVA) ≥ 20/25 in both eyes (0.1 LogMAR); (6) the corneal meridian maximum and minimum values could not fluctuate by more than 10 D; and (7) a difference ≤ 0.50 D in the keratometry between two tests with a minimum period of seven days for contact lens wearers. Amongst the exclusion criteria: (8) eye diseases, such keratoconus or presumed keratoconus; (9) ocular surface pathologies (moderate to severe dry eye, blepharitis and meibomian gland dysfunction, allergic eye diseases, or chemical and thermal burns); (11) retinal vascular pathology signs; (12) connective tissue ailments; (13) prenatal or lactating subjects; (14) nystagmus or strabismus.

### 2.4. Preoperative Examinations

Before the TPRK with PRGF surgical procedure, a complete preoperative examination was done. Patients on soft contact lenses discontinued their use for a minimum period of fourteen days and those with hard contact lenses, four weeks. The test was achieved by an optician-optometrist. Primary variables were uncorrected distance visual acuity (UDVA) and CDVA (expressed in Snellen scale and LogMAR), manifest refraction without and with cycloplegic, within the highest positive refraction method. Astigmatism was assessed by the Jackson cross cylinder procedure. These data were validated with the Wavefront Supported Custom Ablation (WASCA) autorefractor-aberrometer (Carl Zeiss Meditec AG, Jena, Germany). Tomography, central corneal thickness, keratometry were evaluated with the Pentacam HR^®^ single rotation Scheimpflug camera (Oculus Optikgeräte GmbH, Wetzlar, Germany). Retina study was measured with the optical coherence tomography (OCT) (Optovue Inc., Fremont, CA, USA). Corneal biomechanics and intraocular pressure were assessed with CORVIS ST^®^ (Oculus Optikgeräte GmbH, Wetzlar, Germany). Finally, an iris identification test was performed with the autorefractor-aberrometer. Pain score was calculated with the visual analogue scale [18] (no pain 0 and highest pain 10). Re-epithelialization time was assessed with the slit-lamp and the day was reported when the contact lens was removed after completing the healing process. Secondary variables were efficacy index (defined as postoperative UDVA/preoperative CDVA), safety index (defined as postoperative CDVA/preoperative CDVA), and finally predictability index (defined as the difference among the achieved and attempted refraction).

### 2.5. Surgical Technique

All surgical procedures were completed by two specialists with knowledge in laser correction (F.A.-A and J.A.-C). Ten minutes before the surgical procedure, the eye was sterilized with 5% povidone-iodine (Betadine; Meda Manufacturing, Bordeaux, France) and a drop of double local anesthetic (tetracaine 0.1% and oxybuprocaine 0.4%) (Alcon Cusí, El Masnou, Barcelona, Spain) was instilled.

In the first step of TPRK, epithelium was removed with the phototherapeutic keratectomy (PTK) software of the MEL 90 excimer laser (Carl Zeiss Meditec AG, Jena, Germany) with a treatment zone of 8.00 mm. The ablation was personalized to each patient (from 45 to 60 µm) with the values obtained by OCT. Central epithelium thickness was measured with the OCT prior to all surgeries.

In TPRK second step, photorefractive kerectomy (PRK) was performed with the same MEL 90 with an optical zone of 7.00 mm. All surgeries were planned to reach emmetropia. If excimer ablation depth was 40 µm or more, a sponge Weck-Cel sponge (Beaver-Visitec International, Inc., Waltham, MA, USA) soaked with mitomycin C was located on the corneal stroma for thirty seconds. The mitomycin swab was removed and the residual bed cleaned with 100 mL saline solution at room temperature. Then, a bandage contact lens of Comfilcon A (Biofinity Energy; CooperVision, Fairport, NY, USA) was inserted. If the contact lens offered excessive movement, it was changed to another contact lens with a smaller radius of curvature (Lotrafilcon A, Air Optix Night and Day Aqua, Alcon, Fort Worth, TX, USA).

### 2.6. PRGF Preparations

PRGF method preparation was as described with Anitua et al. [14]. Blood samples were collected from every patient using PRGF-Endoret ^®^ Tubes (BTI Biotechnology Institute, Miñano, Spain) containing 0.2 mL of sodium citrate. The blood samples were centrifugated for 8 min in a PRGF-Endoret System centrifuge (BTI Biotechnology Institute, Miñano, Spain) at room temperature. The milliliter above the buffy coat called platelet enriched fraction was extracted. This step is crucial in order to not extract the leucocyte layer. The extraction was incubated with PRGF-Endoret activator (BTI Biotechnology Institute, Miñano, Spain) for one hour at 37 °C. The growth factor enriched was collected by aspiration and next filtered with a 0.2 µm filter. The final preparation was stored at −80 °C until use.

### 2.7. Postoperative Evaluation

PRGF were employed three times every day for the period of the first six weeks [15]. Tobramycin 0.3% and dexamethasone 0.1% (Tobradex; Alcon Cusí, Barcelona, Spain) were employed five times each day for the first week, then fluorometholone 0.3% (FML; Allergan, Westport, Ireland) was employed three times daily for the second week, and finally one time daily for the third week. Patients were observed at 1, 3, 7, 15, 30, and 90 days. A slit-lamp examination, spherocylindrical refraction, and monocular visual acuity were performed in all appointments.

### 2.8. Statistical Analysis

Statistical analysis was supported with SPSS statistics 26.0 (IBM Corporation, Armonk, NY, USA). All visual acuity data were converted into Snellen formats according to standard refractive surgery methodology. Standard graphs for laser and intraocular graph refractive surgery was prepared according to Waring III et al. [19] Statistically significant differences were assessed with the T student test or U of Mann–Whitney according to parametric or not parametric variables. Pain score variable was assessed with repeated measured ANOVA. All statistical tests were performed with a 95% confidence level (*p* < 0.05).

## 3. Results

Patients mean age was 30.17 ± 5.20 (24 to 39) years. In the preoperative, mean sphere was −2.27 ± 1.16 (−1.00 to −5.50) D, mean cylinder refraction was −0.81 ± 0.53 (0.00 to −2.50) D, and mean spherical equivalent refraction was −2.67 ± 1.25 (−1.37 to −6.75) D. Visual acuity data were expressed in Snellen scale (20/x) and LogMAR. Preoperative UDVA was 20/93.33 ± 20/55.93 (20/40 to 20/200), 0.60 ± 0.23 LogMAR. Preoperative CDVA was 20/20, 0.00 ± 0.00 LogMAR in all eyes.

**Figure 1:**
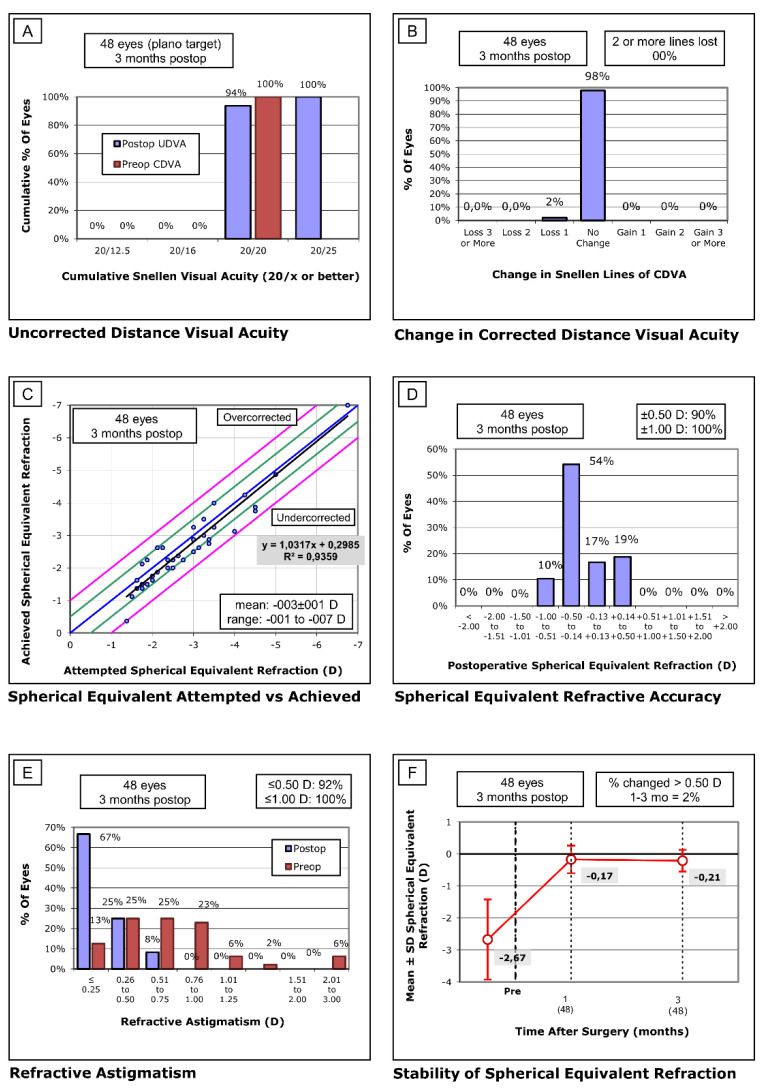
Standard graphs for reporting refractive surgery. (**A**) Uncorrected visual distance acuity (UDVA)–efficacy histogram. (**B**) Change in corrected distance visual acuity (CDVA)–safety histogram. (**C**) Spherical equivalent attempted versus achieved. (**D**) Spherical equivalent refractive accuracy. (**E**) Refractive astigmatism. C–E graphs represent predictability. (**F**) Stability of spherical equivalent refraction.

Postoperative UDVA was 20/20.31 ± 20/1.22 (20/20 to 20/25), 0.00 ± 0.00 LogMAR. Distance cumulative visual acuity (20/x or better) is presented in Figure 1A. At the postoperative follow-up, 94% of eyes achieved 20/20 (0.00 LogMAR) and all eyes achieved 20/25 (0.1 LogMAR). Efficacy index was 1.01 with the plano target. Regarding safety, at the three-months post operation, 98% of eyes did not change CDVA lines. two percent of eyes lost one line of CDVA (Figure 1B). Intraoperative or postoperative complications were absent. Safety index was 1.02. Regarding predictability, achieved refraction versus attempted refraction is presented in Figure 1C. Predictability index was 1.03x + 0.29. Points above blue line represent overcorrection refraction and points under the blue line represent undercorrection refraction. The green line represents a ± 0.50 D and the purple line represent a ±1.00 D. Percent of eyes in postoperative refraction is presented in Figure 1D. In terms of spherical equivalent refractive accuracy, 90% of eyes were within ±0.50 D and all eyes were within ±1.00 D. Percent of eyes with postoperative astigmatism is presented in Figure 1E. Regarding refractive astigmatism, 92% of eyes were ≤0.50 D and 100% of eyes were ≤1.00 D. Finally, stability with preoperative spherical equivalent was −2.67 ± 1.37 D and after three months improved to −0.21 ± 0.34 D, 2% of eyes were ≥0.50 D (Figure 1F). All patients were compliant with their appointments. The main pain score was 3.29 ± 0.61 (3 to 6) score points at day one after the surgeries, then, improved to 0.92 ± 0.57 (0 to 3) score points at day three and finally was 0.08 ± 0.27 (0 to 1) score points at day seven. Wilk’s Lambda was 0.042 (*p* < 0.01), All pain scores pairwise comparisons by repeated measures through ANOVA were statistically significant (*p* < 0.01). The mean time to epithelial healing was 2.50 ± 1.20 (1 to 5) days. No deposits of PRGF were observed on the surface of the bandage contact lens. No patients reported complications or difficulties with the PRGF administration.

## 4. Discussion

Our retrospective study reported visual and refractive outcomes obtained with TPRK and PRGF with MEL 90 excimer laser. We reported, efficacy, safety, predictability, stability, pain score, and re-epithelialization time. To the best of our knowledge, there has not been enough published research with regard to TPRK with PRGF in humans [13].

Regarding efficacy, we documented that 94% reached 20/20 or better UDVA. Clinch et al. [20] in a prospective randomized controlled study, first described classic double-step TPRK results. In 1999, they used a PTK followed by PRK. Our efficacy index was 1.01, better than other double-step TPRK previously reported [21,22,23]. TPRK with double-step revealed comparable efficacy outcomes with excimer laser or mechanical PRK [21,22]. Double-step laser platforms used in previous studies [20,21,22,23] were different from the platform in this study. Additionally, none of the previous studies used PRGF. An interesting point by Shapira et al. [23] was that they found a decrease in the efficacy index. During the time interval of one to three months reported 0.96 and over the next three months it fell to 0.88. In terms of safety, our results showed that no eye lost two lines or more, while only one eye lost a CDVA line. Our safety index was 1.00. Shapira et al. [23] was the only author that reported the safety index in double-step TPRK and obtained 0.99 at three months of follow-up without the use of PRGF in the postoperative period. This TPRK safety index is superior to the efficacy index of conventional PRK or alcohol assistance PRK [24,25]. In our study, no complications were found. A recent review by Abid-Moghaddam et al. [26] showed that the complications and risks after TPRK were lower than manual debridement or alcohol assisted PRK modalities. Data of comparison with other techniques are missing. The most common situations after PRK are postoperative pain and corneal reepithelialization delayed time [26]. These two aspects will be discussed later independently.

Regarding predictability, our results obtained 1.03x + 0.29 (R^2^ = 0.93). Overcorrection or undercorrection may occur during TPRK as well as other corneal surface refractive laser surgeries [21,27,28]. None of the authors analyzing double-step TPRK reported predictability results. Among the authors who analyzed the single-step TPRK [24,27,28,29,30], they obtained predictable results comparable to those reported in our study. Our spherical equivalent refractive accuracy showed that 90% of eyes were in ±0.50 D and all eyes were in ±1.00 D. We found similar results in refractive astigmatism accuracy, 92% ≤ 0.50 D and 100% ≤ 1.00 D. Clinch et al. [20] found comparable spherical accuracy results at three months follow-up with 74.4% of eyes within ±0.50 D and 93.5% of eyes ±1.00 D. Lee et al. [21] reported worse results with only 56.9% of eyes within ±0.50 D or less. Shapira et al. [23] also found less accuracy, when analyzed more than one year postoperatively, with fewer than half of eyes in the TPRK group achieved manifest correction within ±0.50 D of the attempted correction.

Pain score, in our study, at the first postoperative day was 3.29 ± 0.61 and it decreased to 0.92 ± 0.57 and 0.08 ± 0.27 at three and seven days, respectively. Our results showed better pain score results than those previously published by Wang et al. [22] who reported 4.43 ± 1.61 on the first day. They found that pain was relieved at fourth postoperative day and healing epithelial was observed between the fourth and the fifth day. In addition, they did not find any statistically significant differences in terms of pain score between the second and third day both in PRK and TPRK group. At three months, Wang and colleagues obtained excellent visual and refractive results with 93.3 and 90% of eyes with UDVA of 20/20 or better in both groups. Clinch et al. [20] and Shapira et al. [23] did not report postoperative pain score after double-step TPRK. Lee et al. [21] evaluated pain score employing three epithelial removing techniques (mechanical, alcohol, and transepithelial). They found that no difference in subjective pain scores between the three groups. They concluded that using laser-assisted subepithelial keratectomy (LASEK) was not better in decreasing postoperative pain. Specifically, on day one, postoperative pain score was 4.71 and at three days it was 3.04. In terms of re-epithelialization time, in the results shown by Lee et al. [21], 3.10 ± 0.88 days were comparable with those obtained in our study 2.50 ± 1.20 days. Lee et al. [21] were the only authors with double-step TPRK who studied epithelialization time. Javaloy et al. [15] did not achieve significant difference using PRP or not on corneal sensitivity after LASIK. Previous studies [31,32] have shown that corneal nerve regeneration was faster with the use of blood derivates as PRP or PRGF. Blood derivatives, such as AS, were mainly made up of many neurotrophic and epithelial growth factors [9,10,33]. Nerve growth factor is one of the most important and it was found in higher quantities in blood derivatives compared to natural human tears. The nerve growth factor induces an injured neuron’s function restoration that helps in corneal nerve regeneration [31]. However, in the results obtained by Akcam et al. [17], the AS reduced pain score and reepithelialization time compared to an artificial tears group. In their study, surgery was alcohol-assisted PRK. PRGF has been previously used in dry eye disease management after LASIK with an improvement of ocular surface disease index score and Schirmer test [12]. Nevertheless, to our knowledge, Anitua et al. [14], the only PRGF study in PRK, was conducted in mice and reported that PRGF improved epithelial corneal cells increase. In addition, PRGF enhanced wound healing after excimer laser photoablation, reducing corneal haze formation. A limitation of the study was the lack of a control group, the retrospective nature of the research, and that it included a relatively small number of patients with short follow-up with both eyes included in the analysis. As a future line of research, randomized clinical trial with PRGF, PRP, AS, and control group in TPRK would improve scientific evidence in this topic.

## 5. Conclusions

Immediate postoperative pain and re-epithelialization time seems to improve with the use of PRGF in TPRK. PRGF in double-step transepithelial photorefractive keratectomy demonstrated, at three-months follow-up, that this excimer laser surgery technique is effective, safe, predictable, and stable. A larger sample, randomized clinical trials and a long-term follow-up is necessary to corroborate the described findings.

## Data Availability

The data presented in this study are available on request from the corresponding author. The data are not publicly available due to part of future research.
